# Urinary Tract Infection in Overactive Bladder: An Update on Pathophysiological Mechanisms

**DOI:** 10.3389/fphys.2022.886782

**Published:** 2022-07-04

**Authors:** Kylie J. Mansfield, Zhuoran Chen, Kate H. Moore, Luke Grundy

**Affiliations:** ^1^ Illawarra Health and Medical Research Institute and School of Medicine, University of Wollongong, Wollongong, NSW, Australia; ^2^ Department of Urogynaecology, St George Hospital, University of New South Wales, Kogarah, NSW, Australia; ^3^ Visceral Pain Research Group, College of Medicine and Public Health, Flinders Health and Medical Research Institute (FHMRI), Flinders University, Bedford Park, SA, Australia; ^4^ Hopwood Centre for Neurobiology, Lifelong Health Theme, South Australian Health and Medical Research Institute (SAHMRI), Adelaide, SA, Australia

**Keywords:** bladder, overactive bladder, urinary tract infection, bacterial cystitis, inflammation, hypersensitivity

## Abstract

Overactive bladder (OAB) is a clinical syndrome defined by urinary urgency, increased daytime urinary frequency and/or nocturia, with or without urinary incontinence, that affects approximately 11% of the western population. OAB is accepted as an idiopathic disorder, and is charactersied clinically in the absence of other organic diseases, including urinary tract infection. Despite this, a growing body of research provides evidence that a significant proportion of OAB patients have active bladder infection. This review discusses the key findings of recent laboratory and clinical studies, providing insight into the relationship between urinary tract infection, bladder inflammation, and the pathophysiology of OAB. We summarise an array of clinical studies that find OAB patients are significantly more likely than control patients to have pathogenic bacteria in their urine and increased bladder inflammation. This review reveals the complex nature of OAB, and highlights key laboratory studies that have begun to unravel how urinary tract infection and bladder inflammation can induce urinary urgency and urinary frequency. The evidence presented in this review supports the concept that urinary tract infection may be an underappreciated contributor to the pathophysiology of some OAB patients.

## Introduction

Overactive bladder (OAB) is a clinical syndrome defined by urinary urgency, increased daytime urinary frequency and/or nocturia, with or without urinary incontinence (Wein and Rovner, 2002). OAB by definition is idiopathic, “a disease of unknown cause” which is only diagnosed in the absence of other organic disease, such as cancer, neurological or structural abnormalities, or urinary tract infection. Based on these criteria, OAB effects approximately 11% of the western population, significantly reducing quality of life (Wein and Rovner, 2002; Coyne et al., 2011; Eapen and Radomski, 2016; Kinsey et al., 2016).

Despite the accepted idiopathic nature of OAB, several studies in the last decade have provided a new aetiological paradigm; low levels of pathogenic bacteriuria (bacteria in urine) and inflammation are found in substantial proportions of OAB patients (Moore et al., 2000; Walsh et al., 2011; Walsh et al., 2011; Walsh and Moore, 2011; Digesu et al., 2013; Khasriya et al., 2013; Moore et al., 2013; Moore and Malykhina, 2014; Reynolds et al., 2016; Chen et al., 2018) and in a significant portion of women with newly diagnosed OAB (Ognenovska et al., 2021). These discoveries have highlighted key questions: Is the pathophysiology of OAB more complicated and multifaceted than has traditionally been proposed? And what is the mechanism whereby chronic low grade bacterial cystitis could promote sensory dysfunction and incontinence?

This review summarises the key findings of recent laboratory and clinical studies which provide further insight into the relationship between chronic bacteriuria and associated inflammatory mediators in OAB patients.

## Clinical Significance of Overactive Bladder

Approximately 11% of women from western countries are diagnosed with OAB based on the clinical symptoms of urinary urgency, urinary frequency, nocturia, and in some cases, urge incontinence (Eapen and Radomski, 2016). In patients over 40 years of age, the prevalence in European countries is 17% (Milsom et al., 2001). These symptoms severely affect quality of life, contributing to significant psycho-social comorbidities including increased incidence and severity of depression, anxiety, and social isolation, which contribute to declining mental and physical health (Coyne et al., 2011; Kinsey et al., 2016). OAB patients also incur significant personal and societal health care costs associated with repeated primary health care visits and reduced professional opportunities (Coyne et al., 2011; Kinsey et al., 2016; Reynolds et al., 2016). The direct and indirect health care costs associated with OAB have been calculated as over $100 Billion per annum in the United States alone (Ganz et al., 2010; Pierce et al., 2015; Reynolds et al., 2016).

## Diagnosis, Treatment, and Pathophysiology of Overactive Bladder

In the typical clinical care pathway, patients presenting with symptoms of urinary urgency, urinary frequency and nocturia often undergo a diagnostic test involving filling the bladder with saline and observing the presence of spasms of the detrusor muscle (*via* intravesical manometry line), which is termed Detrusor Overactivity. In the absence of organic disease, the condition is termed “Idiopathic,” which is predominant in women but quite uncommon in men. Therefore, while the pathophysiological consequence of the disease is defined by the cystometry testing (i.e. detrusor overactivity), the aetiology underlying this condition remains unknown.

As the chief symptoms of OAB are related to sensory dysfunction, it is logical that the mechanisms underlying OAB are likely related to changes in neuronal excitability and/or exaggerated neuronal firing during bladder filling. This hypothesis is well supported by investigations conducted using animal models of bladder dysfunction (Yoshimura et al., 2014; Grundy et al., 2019). Despite this, OAB patients are initially treated with anticholinergic medications which reduce detrusor muscle contractions and improve bladder capacity, thus lessening their incontinence/frequency/urgency of micturition (Moore and Malykhina, 2014). Unsurprisingly, with little fundamental basis in pathophysiology, these treatments have only limited benefits over placebo and poor continuation rates (Wagg et al., 2012; Kim and Lee, 2016; Yeowell et al., 2018). Our longitudinal study revealed that after 8 years, only 20% of patients treated with antimuscarinic agents have long-lasting improvement (Morris et al., 2008). Unfortunately, about 30%–40% of patients do not respond to these medicines at all. Such non-responsive patients are then denoted as having “Refractory OAB” (Moore and Malykhina, 2014). For these refractory patients, the absence of a defined pathology guarantees patients undergo a diagnostic odyssey, incorporating multiple invasive and non-invasive tests, and numerous prescriptions in an effort to exclude various pathophysiology (Gormley et al., 2015). Such “Refractory” patients may suffer lifelong debilitating symptoms, placing a significant burden on both the patient and the health system.

The large proportion of patients refractory to pharmacotherapy also supports an alternative pathophysiology to detrusor overactivity seen in OAB. Indeed, the relative success of neurotoxins that inhibit sensory nerve activity, such as resiniferatoxin and BOTOX (Guo et al., 2013; Hsieh et al., 2016; Cui et al., 2021), in relieving OAB symptoms refractory to pharmacotherapy suggests increased sensory outflow is responsible for the symptoms of urgency and urinary frequency. However, BOTOX treatment is invasive, has been associated with frequent side effects, and requires repeated doses at approximately 6 months intervals (Schurch et al., 2005; Apostolidis et al., 2009; Chen and Kuo, 2020) indicating the treatment is masking, rather than treating the underlying cause of bladder sensory neuron hypersensitivity.

The initiating or contributing factors in the development of neuronal hypersensitivity in OAB remain to be determined. Numerous aetiologies have been proposed, including altered bladder permeability, inflammation, cross-organ sensitisation, and dysregulation of spinal and/or cortical networks (de Groat et al., 2015; Grundy et al., 2018a). Amongst these potential mechanisms, there is mounting evidence that chronic bacterial infection of the bladder may contribute to the exacerbation of OAB symptoms in susceptible populations *via* direct or indirect sensitisation of sensory neurons (Moore et al., 2000; Walsh C et al., 2011; Brierley et al., 2020).

## Urinary Tract Infections

### Prevalence and Diagnosis

**FIGURE 1:**
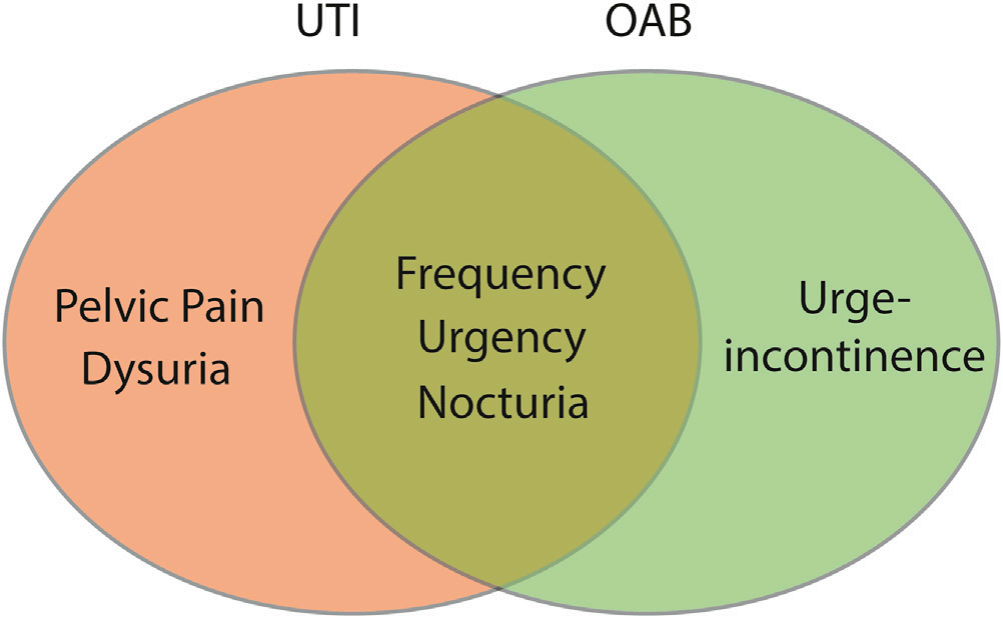
The primary symptoms of OAB and UTI overlap. Urinary frequency, urinary urgency, and nocturia are key symptoms associated with both OAB and acute UTI. Clinically, OAB is differentiated from acute UTI by the absence of bacteriuria and/or a negative dip-stick test.

A urinary tract infection (UTI) is an infection in any part of the upper or lower urinary tract, including kidneys (e.g., pyelonephritis), ureters, bladder, and urethra (Smelov et al., 2016). UTI’s are amongst the most common bacterial infections in the world, affecting more than 150 million people annually worldwide (Smelov et al., 2016; Tandogdu and Wagenlehner, 2016). Despite this prevalence, the vast majority of these infections are caused by a limited number of bacterial species, with *Escherichia coli (E. coli)* that exhibit evolutionary specialisations (Uropathogenic *E. coli,* UPEC) being the stereotypical species (Smelov et al., 2016). UTI’s occur most frequently in the lower urinary tract and typically present with some or all of the following symptoms: dysuria (painful urination), urgency, frequency, and pelvic pain (Chu and Lowder, 2018). These symptoms overlap significantly with those observed in OAB, making exclusion of a UTI a key component in OAB diagnosis (Figure 1).

UTI’s are typically diagnosed in general practice using a urine dipstick as an indirect measure to identify white blood cells (pyuria), production of nitrates in the urine typical of bacteriuria, and the presence of microscopic haematuria as evidence of severe inflammation. If the dipstick test is positive (or symptoms are clearly suggestive) then a fresh unspun midstream (MSU) sample is sent for microscopy to identify 10 or more white blood cells (wbc)/μl (pyuria). The urine is then cultured, and “classical” UTI is diagnosed as greater than 10^5^ CFU of bacteria/ml (Smelov et al., 2016; Tandogdu and Wagenlehner, 2016; Chu and Lowder, 2018). The relative sensitivity and accuracy of these tests is thus a key factor in determining what is a genuine UTI, and determining diagnosis and subsequent treatment.

Ever since the 1950s, when pyelonephritis was a frequent cause of mortality, the threshold of 10^5^ CFU/ml from MSU samples was widely accepted as a cut off to diagnose “Classical UTI” (Chu and Lowder, 2018). However, whilst a cut-off of 10^5^ CFU/ml from MSU will accurately confirm bladder infection, several studies have identified limitations of this stringent criterion as regards bacterial cystitis, i.e., significant lack of sensitivity. Around 50% of patients who present with symptoms of acute UTI that respond positively to antibiotic treatment are mis-diagnosed as “normal” using the 10^5^ CFU/ml cut-off (Stamm et al., 1982; Stark and Maki, 1984). A fine balance must be made, however, as having the criteria too low risks false positives, incorrect diagnosis, and the unnecessary prescription of antibiotics. A criterion of ≥10^2^ or 10^3^ CFU/ml from MSU have been identified as the threshold that resulted in optimal sensitivity and specificity, accurately detecting the majority of genuine infections with low false-positive rates (Stamm et al., 1982; Kunin et al., 1993; Price et al., 2016).

Using this lower criterium of ≥10^2−3^ CFU/ml from MSU, however, revealed that a proportion of patients diagnosed with OAB may have low-level bacterial infection that was previously missed by routine “classical” testing methods (Walsh et al., 2011; Walsh et al., 2011; Walsh and Moore, 2011; Moore and Malykhina, 2014). This has profound implications for effective clinical treatment.

## The Emerging Role of Urinary Tract Infection in Overactive Bladder

### Bacteriuria in Overactive Bladder

Even when using a conventional 10^5^ CFU/ml MSU culture, 6%–17% of women diagnosed with OAB have been found to have a UTI, compared to just 0.5%–2% of women from control groups (Moore et al., 2000; Khasriya et al., 2013; Gill et al., 2021). Similar results have been obtained from MSU samples taken specifically during symptom flares of refractory OAB patients, with 17% of patients versus 2% of the control group showing positive cultures at 10^5^ CFU/ml (Walsh et al., 2011). Interestingly, when both standard (10^5^ CFU/ml) and low-count criteria (10^2−3^ CFU/ml) are used in samples from the same patient cohort, the proportion of those positive for bacteriuria increases (from 17% to 39%), compared to those of a control group (from 2% to 6%) (Walsh et al., 2011) showing that low-count MSU culture may reveal genuine infections in OAB patients that would otherwise be overlooked. Enhanced culture techniques have also revealed higher rates of infection that were previously missed by routine culture, identifying 23% of patients with OAB as positive for UTI, vs. 10% of controls, in a prospective blinded case control study (Khan et al., 2021).

Analysis of catheter (CSU), rather than mid-stream specimens of urine revealed similar results, with the proportion of OAB patients with positive cultures rising from 15% at 10^5^ CFU/ml to 21% at 10^2^ CFU/ml (Khasriya et al., 2010). Two additional studies support the concept that OAB patients have low-count bacteriuria but did not compare directly to an appropriate control group, including a follow up study that investigated catheter rather than MSU specimens from patients with refractory OAB (Walsh et al., 2013). Despite the lack of controls, these studies report similar proportions (27%–29%) of OAB patients with bacteriuria when using the low-count threshold (Hessdoerfer et al., 2011; Walsh et al., 2013).

### Intracellular Bacterial Colonies in Overactive Bladder

Whilst low-count bacteriuria can be quite easily identified by reducing the CFU threshold upon culture, a growing number of studies have shown that uropathogenic *E. coli* invade urothelial cells and form intracellular bacterial communities (IBC’s). A variety of studies have since identified large numbers of bacteria undetected in routine MSU or CSU specimens within cultures from bladder biopsies or shed urothelial cells from OAB patients examined by immunohistochemistry or confocal microscopy of centrifuged/cytospin specimens (Rosen et al., 2007; Vijaya et al., 2013a; Khasriya et al., 2013; Cheng et al., 2016; Gill et al., 2018). However, intracellular bacteria have also routinely been found in large numbers of asymptomatic, or control patients (Khasriya et al., 2013; Cheng et al., 2016), suggesting that the presence of IBC’s alone is not enough to accurately differentiate OAB and control patients. As such, it may not be the simple identification of IBC’s in urine, but a more nuanced diagnostic marker that reveals an accurate distinction between genuine and asymptomatic infection. Our own detailed analysis of exfoliated urothelial cell samples obtained from the urine of patients with OAB or controls revealed filamentous bacteria were significantly more common in patients with OAB (Cheng et al., 2016). Filamentous bacteria are associated with intracellular bacterial growth and bacteria fluxing out of the urothelial cells to recolonise the bladder (Justice et al., 2006). In this context, *E. coli*, the species of bacteria most commonly implicated in UTIs, was found more closely associated with urothelial cells from sediment cultures (*via* confocal microscopy) only in OAB patient samples (Khasriya et al., 2013). Similarly, our further study of urothelial cells obtained from OAB patients demonstrated that high- but not low-density intracellular bacteria correlate with OAB symptom severity, measured by leakage on pad test, leaks per day, and voids per day (Ognenovska et al., 2021).

The contribution of IBC’s to UTI pathophysiology is an emerging field of research. These initial reports suggest that IBC’s within the urothelium may also be an underappreciated component in the symptomology of OAB. Importantly, these bacteria are unlikely to be picked up by increasing the sensitivity of MSU or CSU culture, raising further questions as to the interpretation of urine culture as the gold standard for ruling out genuine UTI’s. The identification and classification of IBC’s is currently impractical for routine clinical practice. However, if the relevance of IBC’s in OAB is confirmed by additional high-quality studies, further urine analysis may be a useful tool in elucidating the pathophysiology underlying the symptoms for OAB patients refractory to traditional treatments.

## Infection Induced Inflammation in Patients With Overactive Bladder

### Clinical Analysis

The relative abundance of low-count bacteriuria and IBCs in urothelial cells isolated from both OAB patients and asymptomatic controls points to the necessity for a distinction between bacterial colonisation, and infection (which is associated with inflammation and host-defence) when determining clinical intervention strategies. One of the challenges we face is that the immune response drives bacteria to localise intracellularly to form IBC’s, where they can evade our traditional host-defence mechanisms. Furthermore, providing distinction between bacterial colonisation and infection will likely have greater relevance going forwards, with the identification of a bladder microbiome consisting of a diverse microbiological flora in the healthy bladder (Neugent et al., 2020).

Pyuria is the clinical standard for identifying localised bladder inflammation in the context of UTI, defined as the presence of 10 or more white blood cells (WBC)/mm^3^ in fresh uncentrifuged urine (Chu and Lowder, 2018). However, pyuria is more commonly determined indirectly *via* the dip-stick test, by detecting leukocyte esterase. As would be expected, the prevalence of pyuria rises with the level of bacteriuria in uncomplicated cystitis and thus, if OAB patients with bacteriuria have genuine bacterial infections, pyuria, and changes in markers of inflammation should be apparent. Indeed, positive routine culture has been shown to be predictive of pyuria in a cohort of OAB patients (Khasriya et al., 2013). OAB patients are significantly more likely than control cohorts to demonstrate a positive dipstick test for leukocytes (39% vs. 9%) (Gill et al., 2021), and 30%–40% of OAB patients have been shown to have pyuria following microscopic analysis of urine (Khasriya et al., 2013; Contreras-Sanz et al., 2016). OAB patients also showed consistently higher microscopic pyuria counts on fresh urine microscopy compared to asymptomatic controls (Gill et al., 2018). Interestingly, pyuria identified by microscopic analysis of urine was recently shown by a group in the United Kingdom to be the most important correlate of symptom severity in OAB, with urgency correlating highly with both pyuria and epithelial cell shedding (Gill et al., 2018), a secondary, and potentially key additional indicator of active infection or inflammation (Mulvey et al., 2001; Klumpp et al., 2006).

### Cytokine Analysis

In addition to immune cells, the inflammatory response to bacteriuria is also mediated by cytokines. Cytokines are essential regulators of both the innate immune response to infection and the inflammatory response to injury, with the concentration of urinary cytokines correlating with symptomatic UTI (Rodhe et al., 2009; Armbruster et al., 2018). Bacteria induced inflammation of the bladder stimulates the release of a large variety of both pro- and anti-inflammatory cytokines and chemokines (Table 1). Some cytokines have been consistently reported to increase during UTI, including IL-1β (Davidoff et al., 1997; Sundac et al., 2016), IL-6 (Davidoff et al., 1997; Tyagi et al., 2010; Sundac et al., 2016), TNFα (Davidoff et al., 1997; Tyagi et al., 2010; Sundac et al., 2016), IL-8 (Tyagi et al., 2010; Hannan et al., 2014; Sundac et al., 2016; Tyagi et al., 2016), and CXCL-10 (Sundac et al., 2016; Tyagi et al., 2016). Similar elevations in the levels of pro-inflammatory cytokines and chemokines have also been reported in murine models of uropathogenic *E. coli* induced UTI (Engel et al., 2006; Hannan et al., 2010; Duell et al., 2012; Tan et al., 2012). This further supports the crucial role of the innate immune system in the bladder response to the presence of bacteria during an acute UTI.

**TABLE 1:** A summary of the literature reports of changes in the levels of urinary cytokines in adult patients with UTI or OAB.

Cytokine	UTI	OAB
*A. Pro-inflammatory cytokines*		
IL1ß	0.005 Davidoff et al. (1997); 0.001 Sundac et al. (2016)	0.023 Chen et al. (2019)
IL2		0.04 Chen et al. (2019)
IL6	0.01 Davidoff et al. (1997); ↓0.01 Tyagi et al. (2010); 0.001 Sundac et al. (2016)	↓0.01 Tyagi et al. (2010); 0.05 Gill et al. (2021)
IL7	0.001 Sundac et al. (2016)	0.07 Chen et al. (2019)
IL9	0.0001 Sundac et al. (2016)	
IL12p_70_	0.0001 Sundac et al. (2016)	↓0.02 Pillalamarri et al. (2018); 0.0002 Chen et al. (2019)
IL17	↓0.014 Tyagi et al. (2010); 0.01 Sundac et al. (2016)	
TNF-α	0.05 Cheng et al. (2016); 0.017 Tyagi et al. (2010); 0.001 Sundac et al. (2016)	0.017 Tyagi et al. (2010) 0.027 Ma et al. (2016), 0.04 Chen et al. (2019)
IFN-γ	0.001 Sundac et al. (2016)	
*B. Chemokines*		
IL8	0.018 Tyagi et al. (2010) 0.05 Hannan et al. (2014) 0.01 Tyagi et al. (2016); 0.001 Sundac et al. (2016)	↓0.01 Tyagi et al. (2016); 0.006 Chen et al. (2019)
CXCL 10	0.01 Tyagi et al. (2016), 0.001 Sundac et al. (2016)	↓0.01 Tyagi et al. (2016); 0.001 Chen et al. (2019)
MCP-1	0.05 Sundac et al. (2016)	0.05 Tyagi et al. (2010); 0.05 Gill et al. (2018); 0.001 Farhan et al. (2019); 0.0001 Ghoniem et al. (2020), 0.04 Chen et al. (2019)
MIP-1α	0.0001 Sundac et al. (2016)	0.05 Tyagi et al. (2010), 0.035 Ma et al. (2016)
MIP-1ß	0.0001 Sundac et al. (2016)	
RANTES	0.05 Tyagi et al. (2010); 0.05 Sundac et al. (2016)	0.034 Chen et al. (2019)
Eotaxin	0.01 Sundac et al. (2016)	
*C. Anti-inflammatory cytokines*		
IL1ra	↓0.05 Tyagi et al. (2016); 0.01 Sundac et al. (2016)	↓0.05 Tyagi et al. (2016)
IL4	0.001 Sundac et al. (2016)	↓0.014 Ma et al. (2016)
IL5		↓0.05 Tyagi et al. (2010)
IL10	0.001 Sundac et al. (2016)	0.05 Tyagi et al. (2010); ↓0.02 Pillalamarri et al. (2018)
IL13		↓0.02 Pillalamarri et al. (2018)

Unless otherwise indicated increased concentrations of cytokines are seen in UTI or OAB patients. ↓ indicates decreased cytokine concentration. p-values are reported in the literature vs. appropriate control patient cohorts.

As a result, numerous studies have investigated urinary cytokines as biomarkers for infection-induced inflammation in OAB patients. In support of the concept that a subpopulation of OAB patients symptoms are driven by an infectious/inflammatory state, a number of studies have shown altered cytokine and chemokine profiles in the urine of patients with OAB (Table 1) (Tyagi et al., 2010; Ghoniem et al., 2011; Tyagi et al., 2014; Ma et al., 2016; Tyagi et al., 2016; Alkis et al., 2017; Pillalamarri et al., 2018). This can manifest as changes in either pro- or anti-inflammatory cytokines, or both. Furthermore, a number of studies have reported that changes in urine cytokine concentration correlates with OAB symptom severity (Smelov et al., 2016; Pillalamarri et al., 2018; Gill et al., 2021), as well as bacterial growth and pyuria count (Gill et al., 2021). In general, however, a large amount of inter-study variability exists in the specific cytokines measured, and the relative changes in urine cytokine concentrations in these OAB patient cohorts. This variability is further compounded by the relatively small number of patients included in these studies. Despite these issues, there is a growing consistency in the evidence for elevations in pro-inflammatory cytokines TNF-α (Tyagi et al., 2010; Ma et al., 2016; Chen et al., 2019), MCP-1 (Tyagi et al., 2010; Chen et al., 2019; Farhan et al., 2019; Ghoniem et al., 2020) and MIP-1 (Tyagi et al., 2010; Ma et al., 2016) as well as reductions in anti-inflammatory cytokines IL-10, IL-1 receptor antagonist, and IL-4 (Ma et al., 2016; Tyagi et al., 2016; Pillalamarri et al., 2018) in OAB patients. A shift in the balance of these cytokines to a more pro-inflammatory state, and a lack of anti-inflammatory cytokines in women with OAB, may allow an inflammatory response to proliferate, thus contributing to the pathogenesis of the disease (Pillalamarri et al., 2018). A thorough, large scale trial, including well characterised patient cohorts is required to determine the true incidence and relative changes in urinary cytokines in OAB patients.

**FIGURE 2:**
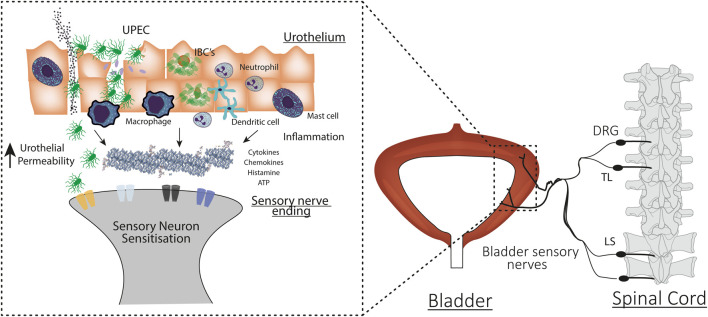
Mechanisms underlying urinary tract infection induced bladder hypersensitivity. Uropathogenic *E. coli* (UPEC) invade urothelial cells during bladder colonisation. UPEC infection evokes an inflammatory response and the recruitment and activation of immune cells. UPEC also form intracellular bacterial colonies (IBCs) to evade the immune system and prolong urothelial colonisation. UPEC infection induces urothelial sloughing and apoptosis, increasing bladder permeability and allowing the toxic contents of the urine and bacteria to access the underlying interstitium. In response to damage, immune cells and the urothelium also release cytokines, chemokines, and neurotransmitters that can bind to receptors and ion channels on the peripheral ends of bladder-innervating sensory neurons. Immune and urothelial cell secretions, bacteria, and urine combine to sensitise the peripheral ends of bladder neurons. Sensitised neurons respond to bladder distension with higher intensity firing. This exaggerated signal travels to the spinal cord and activates second order neurons that travel to the brainstem and brain to induce exaggerated bladder sensation.

Considerable evidence is emerging from experiments in rodents indicating that hyperexcitability of bladder-innervating sensory neurons is triggered by bladder inflammation and may play a critical role in the pathogenesis of OAB (Grundy et al., 2018a; Geppetti et al., 2008). As described in Figure 2, the myriad molecular products of inflammation are able to act as potent neuromodulators, increasing the excitability of sensory neurons to physiological stimuli in a wide variety of organs (Pinho-Ribeiro et al., 2017). Evidence for inflammatory mediators increasing the excitability of bladder innervating sensory neurons, and the importance of inflammation in translating urinary tract infection into the symptoms of urgency and frequency experienced in patients with OAB, will be discussed in detail below.

## Translation of Infection and Inflammation Into Overactive Bladder Symptoms

### Bladder Sensation

Bladder sensation is initiated *via* the activation of primary sensory neurons embedded throughout the bladder wall, innervating both the detrusor smooth muscle and urothelium (Spencer et al., 2018). Sensory signals are generated by the activation of mechanosensory ion channels on bladder-innervating neurons during bladder stretch. This sensory information is relayed *via* synapses within the spinal cord dorsal horn to the brainstem or thalamus (Fowler et al., 2008). The intensity of this sensory signal is proportional to the degree of bladder stretch, leading to the progression of bladder sensations from fulness, to urge, discomfort and finally pain (Fowler et al., 2008; Grundy et al., 2018b). An increase in the intensity of this sensory afferent signal thus leads to exaggerated sensation at lower bladder volumes.

### Mechanisms of Infection/Inflammation Induced Hypersensitivity

The penetration of bladder-innervating sensory neurons into the sub-urothelium and urothelium provides the underlying physiological architecture to allow urinary tract infection and inflammation to induce the symptoms of OAB (Spencer et al., 2018). A number of mouse studies have shown that various inflammatory conditions and inflammatory mediators increase the excitability of bladder innervating sensory neurons (de Groat and Yoshimura, 2009; Grundy et al., 2020a). Furthermore, and as described above, it is well established that acute urinary tract infection evokes a potent inflammatory response in mice, consisting of a significant increase in cytokines and other secreted factors (Abraham and Miao, 2015; Brierley et al., 2020).

In order to test the hypothesis that UTI induced inflammation causes exaggerated bladder sensory signalling, our recent study investigated how inflammatory mediators released during acute UTI in mice altered bladder sensory nerve activity to distension utilising an established *ex-vivo* bladder afferent recording model (Grundy et al., 2018b; Grundy et al., 2020a; Grundy et al., 2020b; Brierley et al., 2020). Pooled inflammatory supernatants containing an array of cytokines were isolated from mice 8 h after infection with uropathogenic *E. coli*. This inflammatory supernatant was then instilled into the bladder lumen of non-infected mice, and both intra-bladder pressure and sensory nerve activity during graded bladder distension were recorded. Through these experiments we were able to show that the inflammatory environment that is generated during UTI is able to sensitise bladder sensory neurons to distension, exaggerating the mechanosensitive response to bladder filling and reducing the activation threshold pressure (Brierley et al., 2020). As such, neurons that usually only respond to noxious levels of bladder distension (high-threshold afferents), now exhibited robust responses to physiological levels of distension in the presence of the UTI supernatant. In addition, previously mechanically insensitive “silent” afferents became mechanosensitive in the presence of inflammatory supernatants, further increasing the overall sensory output from the bladder. These experiments in rodents provide key mechanistic insights into the molecular interactions that may mediate the development of infection induced bladder hypersensitivity in patients. The real life significance of these molecular interactions is that hyperexcitability of bladder sensory pathways translates to lower perception thresholds, which may generate the primary symptoms of OAB seen in patients, including urgency, frequency, and nocturia (Parsons and Drake, 2011; Yoshimura et al., 2014; Grundy et al., 2020a; Grundy et al., 2020b; Brierley et al., 2020).

It is also possible that bacteria are able to directly and indirectly sensitise bladder-innervating sensory neurons. Bacteria can activate sensory neurons that modulate pain in the skin (Chiu et al., 2013). Furthermore, the toxins and metabolites released during bacterial growth and invasion are able to sensitise neurons, as well as in-directly activate neurons and evoke pain (Chiu et al., 2013; Yang and Chiu, 2017; Blake et al., 2018; Uhlig et al., 2020). This has recently been explored *in vitro* using the cell bodies of bladder-innervating sensory neurons isolated from the dorsal root ganglia (DRG) of mice (Montalbetti et al., 2022). In these experiments, both LPS and the virulence factors produced by UPEC during growth were able to sensitise subsets of bladder-innervating sensory neurons to enhance their excitability, representing an additional mechanism whereby infection may be able to evoke hypersensitive bladder symptoms.

### Indirect Mechanisms

As well as directly activating bladder afferent nerves, bladder infection also promotes urothelial shedding, a host-defence mechanism that rapidly removes infected urothelial cells from the bladder wall (Rosen et al., 2007; Smith et al., 2008; Thumbikat et al., 2009; Wood et al., 2012). Whilst effective in preventing bacteria establishing a foothold within the urothelium, this mechanism transiently increases bladder permeability until the infection is cleared and injury-induced proliferation of urothelial cells restores urothelial barrier integrity (Mysorekar et al., 2009; Shin et al., 2011). Increased urothelial permeability provides an opportunity for the toxic contents of the urine, that are usually sequestered in the bladder lumen, to access underlying sensory nerves. In our model of experimentally induced bladder permeability in mice we have been able to show increased urothelial permeability induces excitability of mechanosensitive bladder afferents (Grundy et al., 2020b), suggesting that increased bladder permeability alone may give rise to bladder sensory symptoms associated with neuronal hyperexcitability.

## Evidence and Implications for Clinical Practice

The information presented in this review poses a number of questions relating to the treatment and diagnosis of OAB. Chief amongst these include the potential use of antibiotics and anti-inflammatory drugs in the treatment of subsets of OAB patients. And could the presence of specific inflammatory mediators or intracellular bacterial colonies in the urine be exploited as a novel diagnostic biomarker for identifying these otherwise overlooked patients?

### Does Antibiotic Treatment of Urinary Tract Infections Relieve Overactive Bladder Symptoms?

There have been two prospective trials of rotating antibiotics for patients with OAB (Khasriya et al., 2011; Vijaya et al., 2013b). The first study demonstrated improvements in urinary leakage and urgency in women treated with prolonged courses of antibiotics in addition to standard anticholinergic therapy (Khasriya et al., 2011). This was followed by a second study confirming similar results in a more refractory OAB group (Vijaya et al., 2013b).

As a result of the promising outcome from the two prospective studies we conducted a placebo controlled randomised trial involving 6 weeks of antibiotics in addition to standard anticholinergic therapy in patients with refractory detrusor overactivity (Chen et al., 2021a). Microbiological data was collected throughout the trial with 10 samples per patient collected over 6 months. This phase IIb trial revealed that antibiotic therapy reduced UTI rates, and corresponded with a clinically significant reduction in urinary incontinence on 24 h pad test (pad weight reduced by 75 g, *p* < 0.008) at 6 months. Improvements were also seen in measures of OAB symptoms such as leaks per day (1 less leak by 6 weeks and 2 less leaks per day by 6 months). In addition, patients who received antibiotics reported improvements on quality of life measures such as ICIQ, and OAB-q. Importantly, this improvement was sustained throughout the next 6 months [significant lower ICIQ score (Mean −5.57)], despite only 6 weeks of antibiotic therapy. Because of the robust collection and analysis of the relevant outcome measures, this study was able to provide objective evidence (i.e., reduced amount of leakage experienced in 24 h) of the biological effect of antibiotics in patients with OAB who were refractory to standard treatments. Based on these findings we concluded that lower rates of UTI experienced by OAB patients treated with antibiotics, and thus lower inflammation, may account for the increased response to anticholinergics in the subsequent months.

In addition to the findings described above, the longitudinal nature of the trial also revealed UTI requiring antibiotic treatment in over 40% of the OAB women who were in the placebo arm. In addition, post antibiotic therapy, breakthrough infections were identified in almost 20% of the women in the antibiotic group. The high rates of symptomatic UTI in the refractory OAB cohort emphasises the importance of UTI to the aetiology of this condition, and supports the proposed paradigm of an infectious subgroup of OAB patients. The trial was halted after interim analysis, due to recruitment issues and ethical concerns raised by the high rates of UTI in these patients.

### Does Antibiotic Treatment of Urinary Tract Infections Reduce Inflammation and Intracellular Bacterial Colonies?

Using samples collected as part of the phase IIb trial described above, we were able to investigate the mechanisms behind the improvement in outcomes in the OAB patients. Our follow-up analysis of the trial cohort revealed that cytokines associated with activation of the innate immune system are reduced by antibiotic therapy in women with OAB (Chen et al., 2021b). In particular, antibiotic treatment was associated with significant reductions of pro-inflammatory cytokines IL-1α, IL-1Ra, IL-6, IL-8, and CXCL-10 and the anti-inflammatory cytokine IL-10, which are all cytokines typically present in UTIs (Sundac et al., 2016). The lower concentrations of the pro-inflammatory cytokines, especially CXCL-10, were associated with lower OAB symptom score, suggesting decreasing the inflammatory response leads to less urgency experienced by OAB patients treated with antibiotics. Other studies also support the hypothesis that changes in cytokine concentrations correlates with symptom severity. For example, Pillalamarri et al. (2018) reported that the pro-inflammatory cytokine IL-1β expression was significantly associated with worsening OAB symptoms (Pillalamarri et al., 2018). A recent longitudinal study showed the anti-inflammatory cytokine IL-6 was found to be significantly higher in OAB patients when compared with controls, which also correlated with both bacterial growth and pyuria count over a 12 month period (Smelov et al., 2016). Another longitudinal study (conducted over 12–14 weeks) reported that at the initial visit MCP-1 levels correlated with symptom severity and that MCP-1 levels decreased in women who responded to treatment (Ghoniem et al., 2020).

As well as affecting release of proinflammatory cytokines, antibiotic treatment was also found to significantly reduced high-density IBC’s in exfoliated urothelial cells from OAB patients (Ognenovska et al., 2021). The decrease in high-density IBC’s significantly correlated with improved symptom scores (reduction in urinary incontinence, decreased leaks per day and decreased voids per day) (Ognenovska et al., 2021)**.** Antibiotic therapy also significantly reduced the likelihood of identifying bacterial filaments in samples from women with OAB (Ognenovska et al., 2021). As mentioned previously, bacterial filaments are associated with intracellular growth of UPEC (Justice et al., 2006). In addition, antibiotic therapy increased the proportion of urothelial cells that were free of bacteria.

Thus, while antibiotic therapy appears to be effective in relieving OAB symptoms, issues surrounding microbial resistance and breakthrough infection suggest that alternative treatments for UTI are warranted. Recently, anti-inflammatory agents such as ibuprofen and indomethacin have been shown to improve symptoms of acute UTI in patients without OAB (Gágyor et al., 2015; Kronenberg et al., 2017). Randomised trials have also shown that ibuprofen given to woman with suspected UTI can reduce dysuria and need for subsequent antibiotics (Moore et al., 2019). Interestingly, two small studies in the 1980s demonstrated that indomethacin significantly improved urge incontinence symptoms in women with OAB (Cardozo and Stanton, 1980; Delaere et al., 1981). However, due to limited understanding of the role of urinary tract infections in OAB at the time, no further studies using these therapies were undertaken.

**FIGURE 3:**
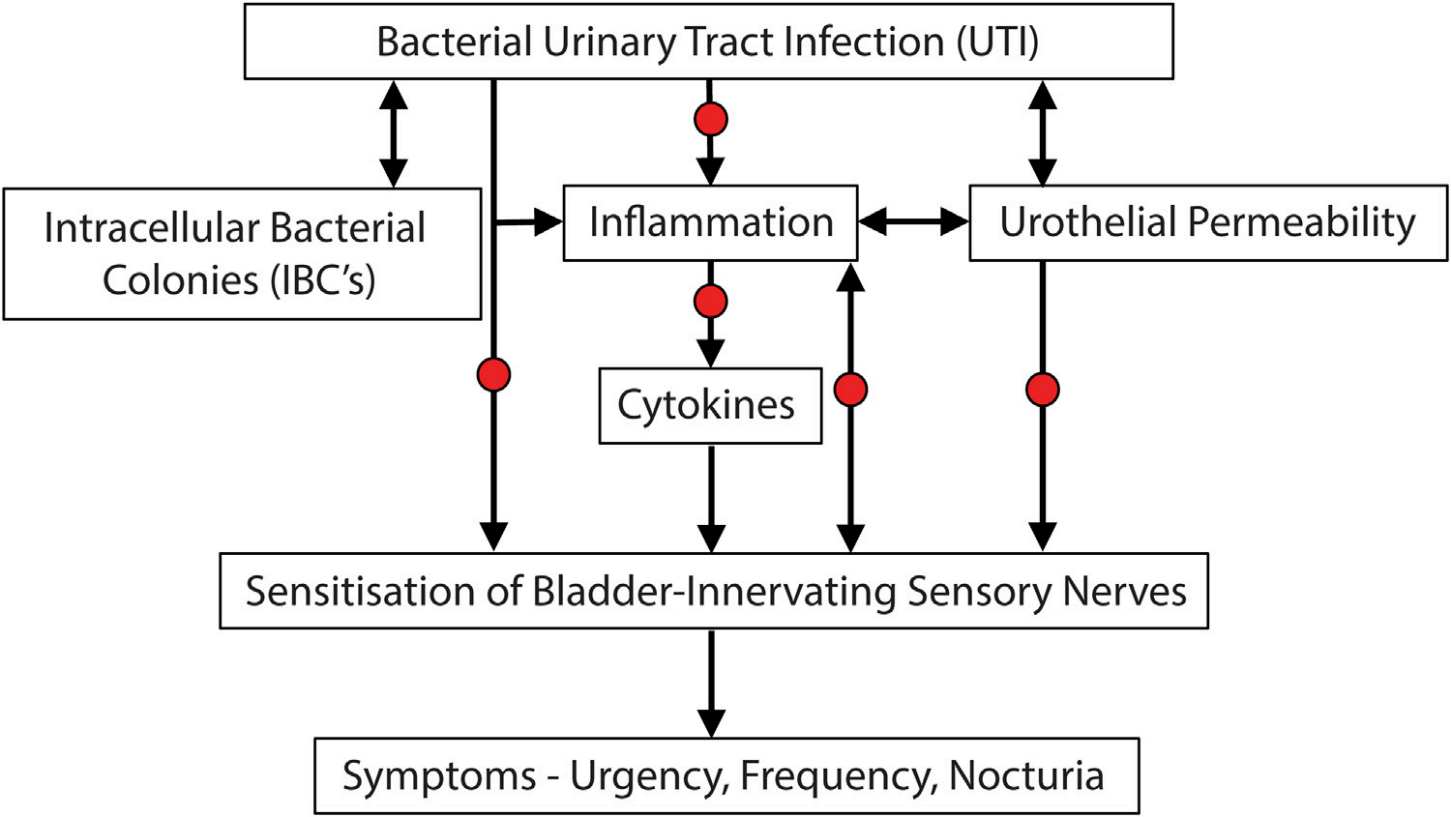
Potential etiological cascade and pathogenesis underlying UTI induced symptoms of bladder hypersensitivity. Evidence is accumulating that subsets of OAB patients may in fact have an underlying bacterial infection, leading to inflammation and the sensitisation of bladder-innervating sensory nerves. Arrows represent causative links. Red dots represent potential opportunities for treatment of UTI induced OAB symptoms.

The studies outlined above have determined that antibiotic therapy leads to improvement in OAB symptoms in women who are refractory to other standard treatments. This improvement in symptoms is associated with a decrease in the urine concentration of pro-inflammatory cytokines and a decrease in the presence of bacteria associated with the urothelium. Combined these studies provide evidence for an inflammatory/infectious aetiology of OAB in women who are refractory to standard therapy. We therefore hypothesise that in women who are refractory to standard therapy, UTI leads to intracellular colonisation of urothelial cells, triggering an inflammatory response (with release of cytokines) and an increase in urothelial permeability. This then sensitises bladder sensory nerves so that they respond more easily to mechanical stretch of the bladder wall. This sensitisation then triggers the symptoms associated with OAB including urgency, frequency and nocturia (Figure 3).

## Conclusion

There is accumulating evidence that we need to revisit the OAB phenotype given the association of the condition with bacterial colonisation of the urothelium. Multiple studies now implicate genuine UTI in the pathogenesis of OAB for a sub-group of patients who are refractory to standard therapy. The detection of bacterial invasion of urothelial cells, and subsequent inflammation, are shown to be key elements in prompting sensitisation of bladder-innervating sensory nerves to bladder distension, which would then evoke the symptoms of urgency, frequency, and nocturia. Small scale trials have shown antibiotics may be a useful tool to treat these patients, however, due to the growing risk of antibiotic resistance, prescription of antibiotics to large cohorts of patients is not desirable. As such, there is a need for alternative therapies to treat the infection and pro-inflammatory state that may include anti-inflammatory agents, or other treatments targeting the microbiome and urothelial environment. In addition, the methods used to diagnose UTI need to be improved. The current threshold values used to diagnose UTI are misleading and the culture techniques used often fail to detect the presence of bacteria. Improvements in our approaches for identifying the presence of bacteria in urine would greatly enhance our capacity to accurately diagnose UTI.
